# The effect of grit on psychological well-being in collegiate athletes: the moderating role of social support

**DOI:** 10.3389/fspor.2025.1711128

**Published:** 2025-11-28

**Authors:** Kyoo-Ho Lee, Kyung-Rok Oh

**Affiliations:** Department of Physical Education, Kyung Hee University, Yongin, Republic of Korea

**Keywords:** collegiate athletes, grit, psychological well-being, social support, mental health, environmental mastery, personal growth, positive relations

## Abstract

**Introduction:**

This study examined the association between grit and psychological well-being among collegiate athletes and investigated whether social support from coaches and teammates moderates this relationship.

**Methods:**

A total of 342 Korean collegiate athletes completed validated measures of grit, perceived social support, and Ryff's psychological well-being dimensions. PROCESS Macro Models 1 and 2 were used to test direct and moderating effects.

**Results:**

Grit significantly predicted autonomy, environmental mastery, personal growth, and positive relations. Coach support attenuated grit's positive effects in several domains, whereas teammate support strengthened grit's influence, particularly for interpersonal well-being.

**Discussion:**

These findings highlight the interplay between personal traits and environmental factors in athletes' mental health. Fostering grit within supportive sport environments may enhance collegiate athletes' psychological well-being.

## 1 Introduction

Collegiate athletes operate in high-pressure environments that pose substantial challenges to mental health. Balancing intensive training and competition with academic demands often elevates stress, undermines psychological well-being, and increases the risk of burnout (1). In response, sport organizations and scholars have increasingly recognized mental health as a critical concern in athletic contexts.

Recent discourse in collegiate sport emphasizes prioritizing the holistic well-being of student-athletes, moving beyond narrow performance outcomes (2). Psychological well-being—encompassing life satisfaction, emotion regulation, and adaptive coping—has emerged as a cornerstone of sustainable athletic performance and long-term development. Accordingly, identifying both personal and environmental resources that support mental health under competitive stress has become a central focus in contemporary sport psychology.

### 1.1 Grit and athlete mental health

Among psychological constructs relevant to performance and well-being, grit has garnered growing scholarly attention. Defined as passion and perseverance toward long-term goals (3), grit enables individuals to remain committed in the face of setbacks and to sustain effort over time. In athletic contexts, grit overlaps conceptually with mental toughness while remaining a distinct construct, reflecting a persistent orientation toward excellence and resilience under pressure (4). Athletes high in grit are more likely to stay engaged, recover from failure, and persist in goal-directed efforts across training and competition cycles (5). Recent evidence indicates that grit is positively associated with both subjective and eudaimonic well-being in student-athletes (6) and negatively associated with burnout (7). Moreover, grit demonstrates incremental predictive validity beyond related traits such as conscientiousness and self-control (8). Collectively, these findings position grit as a distinct motivational resource that contributes not only to athletic performance but also to the promotion of mental health in competitive sport environments. From a self-determination perspective, grit can be viewed as a form of autonomous motivation that reflects the internalization of long-term goal pursuit (9). Rather than being driven by external rewards or social pressure, high-grit athletes persist because their goals are personally meaningful and self-endorsed. This perseverance satisfies the basic psychological needs for autonomy and competence, thereby linking grit to higher levels of well-being. However, when persistence stems from externally imposed expectations or controlling environments, grit may lose its adaptive quality, leading to overtraining or diminished enjoyment. Thus, grit's influence on athlete mental health depends heavily on the surrounding motivational climate.

### 1.2 Psychological well-being in athletes

Psychological well-being refers to optimal psychological functioning and encompasses autonomy, environmental mastery, personal growth, and positive relations (34). Among athletes, higher well-being is associated with sustained motivation, emotion regulation, and resilience—factors that promote sport engagement and overall life satisfaction. Conversely, diminished well-being is linked to greater distress, anxiety, and burnout, particularly among collegiate athletes who must navigate dual roles and performance-related stressors (1). Empirical work also shows that mental well-being enhances concentration, perseverance, and adaptive coping in competitive sport contexts (10). According to Self-Determination Theory (11), psychological well-being arises when individuals experience satisfaction of the three basic psychological needs—autonomy (a sense of volition and self-endorsement), competence (a sense of mastery and effectiveness), and relatedness (a sense of connection and belonging). For athletes, grit may enhance autonomy and competence by sustaining effort toward self-concordant goals, yet well-being also depends on relatedness, which is often shaped by the quality of social support within their sport environment. Accordingly, identifying personal psychological resources—such as grit—that support well-being under high-pressure athletic conditions remains a central focus for future research.

### 1.3 The role of social support

While personal traits such as grit are essential, environmental resources—particularly social support—are likewise influential in shaping psychological well-being. Social support encompasses emotional, informational, and instrumental assistance provided by individuals within an athlete's network, including coaches and teammates. According to the stress-buffering model (12), social support protects individuals from the harmful effects of stress, thereby preserving mental health. In sport contexts, both perceived and received support have been linked to reduced stress and improved psychological functioning (13). Coaches offer encouragement, constructive feedback, and mentorship that enhance athlete satisfaction and emotional stability (14). Similarly, peer support fosters belonging and shared understanding, mitigating loneliness and reinforcing adaptive coping (35). Notably, social support may moderate the influence of personal traits—such as grit—on psychological outcomes. Athletes high in grit may derive greater psychological benefits when embedded in supportive environments. However, empirical evidence on these moderating effects remains limited, particularly across diverse cultural contexts, underscoring the need for further investigation. From a motivational standpoint, social support can either facilitate or frustrate these basic needs. Autonomy-supportive coaching—characterized by empathy, choice, and acknowledgment of athletes' perspectives—enhances intrinsic motivation and reinforces grit's positive effects. In contrast, controlling or overly directive support may undermine athletes' sense of autonomy, leading to dependence on external validation and weakening the benefits of grit. This distinction suggests that social support is not uniformly beneficial; its impact depends on whether it is perceived as empowering or controlling. Accordingly, this study anticipates that coach support may attenuate, and teammate support may amplify, grit's positive association with well-being.

### 1.4 Rationale for the present study

Despite growing scholarly interest in grit and mental health, most empirical work has been conducted in Western contexts. Evidence remains scarce on how these processes manifest in East Asian settings such as South Korea, where unique training environments, collectivist cultural values, and hierarchical coach–athlete dynamics may shape both the expression of grit and access to social support (36). Korean collegiate athletes often face demanding schedules and elevated performance expectations that may distinctly affect their psychological functioning. However, few studies have examined the association between grit and psychological well-being in this cultural context, and even fewer have explored the moderating role of social support. Addressing this gap holds both theoretical and practical significance: it contributes to the cross-cultural validation of grit and informs the development of athlete-support strategies in Korean university sport systems. Building upon Self-Determination Theory (SDT; 9, 11), grit can be conceptualized as a form of autonomous motivation that reflects the internalization of long-term goal pursuit. High-grit athletes persist not merely because of external rewards or pressures but because their goals are personally meaningful and self-endorsed—thereby satisfying the basic psychological needs for autonomy and competence. Recent empirical evidence also supports this motivational pathway. Mindfulness-based psychological training has been shown to enhance athletes' achievement motivation, self-confidence, and performance outcomes, which conceptually parallels and reinforces the mediating role of self-efficacy proposed in the present model (15).

Within this framework, social support functions as an environmental condition that can either facilitate or frustrate these needs depending on its quality. Autonomy-supportive coaching reinforces grit's benefits by validating athletes' perspectives and encouraging volitional engagement, whereas controlling or overly directive support may undermine grit's self-regulatory nature by reducing perceived autonomy and fostering dependence on external validation. Accordingly, the current study posits that the relationship between grit and psychological well-being will be strengthened under supportive peer climates but weakened under excessively controlling coach support. This integrative model bridges SDT and the stress-buffering perspective, positioning grit as an internally regulated motivational resource whose adaptive potential depends on the autonomy-supportiveness of the social environment. Furthermore, the Korean sport context presents unique cultural dynamics that may shape how grit and social support function. Rooted in Confucian hierarchical and collectivist traditions, Korean athletes are often socialized to value obedience, conformity, and interdependence over personal autonomy. Within such contexts, “autonomy” may not signify independence from others—as conceptualized in Western SDT literature—but rather a sense of volitional engagement within relational obligations. Similarly, “coach support” may be interpreted not only as encouragement but also as directional authority that reinforces hierarchical norms. Consequently, high levels of coach involvement could be perceived as controlling rather than autonomy-supportive, especially for athletes who internalize effort and perseverance as self-driven virtues. This cultural nuance helps explain why excessive or directive coach support might attenuate the positive effects of grit on well-being in the current study. Integrating cultural perspectives into the SDT framework thus provides a more context-sensitive understanding of how motivational processes operate within East Asian sport systems. It is also important to note that Ryff's (34) conceptualization of autonomy emerged within Western individualistic paradigms, emphasizing independence and personal control. By contrast, this study situates autonomy within a collectivist cultural framework, proposing that psychological well-being in Korean athletes reflects relational autonomy—the capacity to act with volition while honoring social and hierarchical interdependence. This reinterpretation not only refines the cross-cultural applicability of Ryff's well-being model but also advances SDT by demonstrating how autonomy can manifest differently across cultural contexts (16).

### 1.5 Hypotheses

Drawing upon self-determination theory (11) and the stress-buffering model (12), this study examined the direct and moderating effects of grit and perceived social support (from coaches and teammates) on psychological well-being among collegiate athletes.

H1. Grit will be positively associated with overall psychological well-being.

H2. Grit will be positively associated with autonomy.

H3. Grit will be positively associated with environmental mastery, personal growth, and positive relations with others.

H4. Perceived coach support will moderate the relationship between grit and psychological well-being such that the positive association will be weakened at higher levels of coach support.

H5. Perceived teammate support will moderate the relationship between grit and psychological well-being such that the positive association will be strengthened at higher levels of teammate support.

H6. The moderating effects of social support (from coaches vs. teammates) will differ across specific well-being domains, particularly in interpersonal-related outcomes.

**Figure 1:**
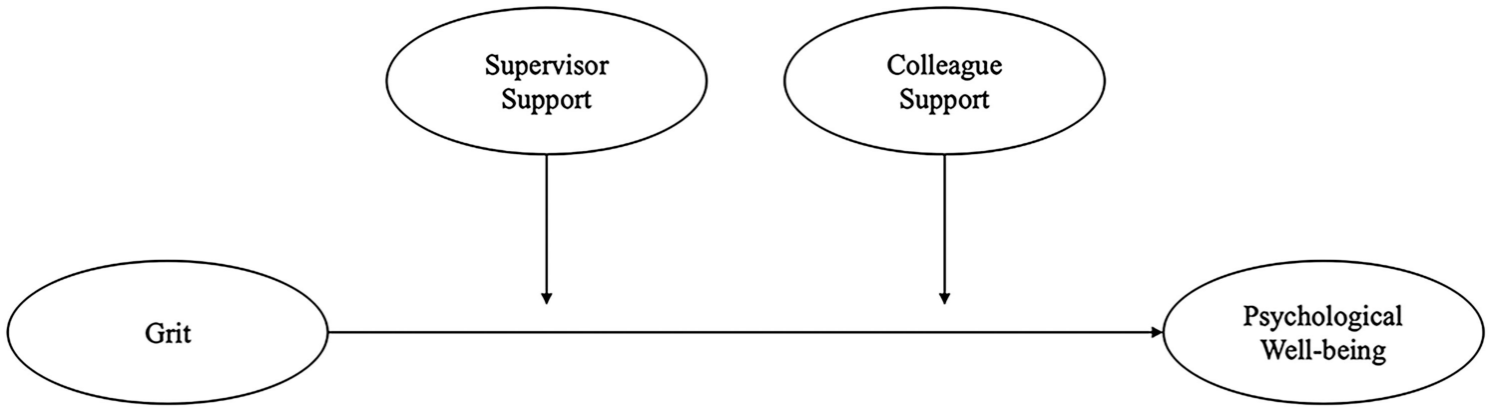
Conceptual model.

The conceptual model of this study is shown in Figure 1.

## 2 Methods

### 2.1 Participants

A total of 342 collegiate athletes (226 males and 116 females; *M* = 21.18, *SD* = 2.94) voluntarily participated in the study. All participants were officially registered under the Korean Sport & Olympic Committee and represented various individual sports (*n* = 175; e.g., track and field, swimming, taekwondo) and team sports (*n* = 167; e.g., soccer, basketball, volleyball). Athletic experience ranged from 1 to over 13 years.

**Inclusion criteria were:** (a) currently enrolled as full-time student-athletes at accredited universities in South Korea; (b) participation in regular collegiate-level training programs supervised by certified coaches; and (c) active competition in official university or national-level events during the data collection period.

**Exclusion criteria were:** (a) athletes who were on medical leave or rehabilitation at the time of data collection; (b) athletes who were not officially registered under the Korean Sport & Olympic Committee; and (c) incomplete or duplicate survey responses. After data screening, 342 valid cases were retained for analysis.

Prior to the main analyses, we examined potential group differences across gender, sport type (individual vs. team), and athletic experience. Independent sample t-tests and ANOVAs revealed no significant differences in grit, social support, or any dimension of psychological well-being across these demographic factors. Given the absence of statistically or practically meaningful variation, these variables were not included as control variables in the final models. This analytic decision is consistent with current methodological recommendations, which suggest that control variables should only be retained when they exhibit significant associations with the main study variables or when strong theoretical justification exists (17).

The study protocol was approved by the Institutional Review Board of Kyung Hee University, Global Campus [KHGIRB-25-396(EA)], and all participants provided written informed consent in accordance with the Declaration of Helsinki.

### 2.2 Measures

All instruments used in this study were previously validated in the domains of sport and health psychology by their original developers—Duckworth et al. (3) for grit, Ryff (34) for psychological well-being, and Zimet et al. (37) for perceived social support—and were culturally adapted for use with Korean collegiate athletes. Each item was assessed using a 5-point Likert scale ranging from 1 (strongly disagree) to 5 (strongly agree). All scales underwent a standard cultural adaptation procedure following international guidelines, including forward–backward translation, expert review, and pilot testing with a sample of collegiate athletes to ensure semantic equivalence and contextual relevance.

#### 2.2.1 Grit

Grit was assessed using the 12-item *Grit Scale* developed by Duckworth et al. (3), which measures individuals' passion and perseverance toward long-term goals. Sample items include “I have overcome setbacks to conquer an important challenge” and “I am diligent.”

#### 2.2.2 Psychological well-being

Psychological well-being was measured using a 21-item scale adapted from Ryff's multidimensional model (34), encompassing four core subdomains relevant to athlete mental health: autonomy (e.g., “I have confidence in my own opinions”), environmental mastery (e.g., “I am good at managing the responsibilities of daily life”), personal growth (e.g., “For me, life has been a continuous process of learning and growth”), and positive relations with others (e.g., “People would describe me as a giving person”). Although self-acceptance was included in the original model, it was excluded from the present study.

#### 2.2.3 Social support

Perceived social support was assessed using a modified version of the Multidimensional Scale of Perceived Social Support (MSPSS; Zimet et al., (37)), adapted for use in the sport context. Three sources of support were measured using four items each: supervisor support (e.g., “There is a special person who is around when I am in need”), colleague or teammate support (e.g., “I have friends with whom I can share my joys and sorrows”), and family support (e.g., “My family really tries to help me”). Family support was not included in the analyses, as it was not tested as a moderator in the present study. All items were contextually adapted to reflect sport-specific interpersonal environments. Descriptive statistics and bivariate correlations are presented in Table 1.

**Table 1:** Correlation matrix, descriptive statistics, and reliability of key variables.

Variables	1	2	3	4	5	6	7
1. Grit	1						
2. Supervisor Support	0.207***	1					
3. Colleague Support	0.525***	0.48***	1				
4. Autonomy	0.653***	0.188***	0.462***	1			
5. Environmental Mastery	0.683***	0.204***	0.533***	0.73***	1		
6. Personal Growth	0.722***	0.214***	0.522***	0.708***	0.713***	1	
7. Positive Relations with Others	0.548***	−0.058	0.105	0.557***	0.462***	0.616***	1
M	3.676	3.740	4.271	4.020	4.073	4.021	3.522
SD	0.448	0.694	0.664	0.704	0.639	0.694	1.229
Reliability	0.623	0.617	0.877	0.674	0.639	0.632	0.901

***Indicates *p* < .001.

## 3 Results

In order to examine the hypothesis, process macro model 1 and 2 were employed. First, process model 2 was implemented to examine whether the two types of social support (i.e., supervisor and colleague) moderate the effect of grit on each dependent variable. Subsequently, additional analysis was conducted using Process Model 1 only when the interaction term was significant. This was an alternative approach to examine conditional moderation effects to clarify each moderation effect. In this analysis, another moderator and the interaction term were controlled. The results of the regression analysis are presented in Table 2.

**Table 2:** The results of the regression analysis about phycological well-being.

Predictors	Autonomy		Environmental mastery		Personal growth		Positive relations with others	
	*b*	*SE*	*b*	*SE*	*b*	*SE*	*b*	*SE*
Constant	−6.023**	1.898	−8.053***	1.588	−5.985***	1.675	−4.820	3.490
Grit	2.742***	0.583	3.332***	0.487	2.679***	0.514	2.987**	1.071
Supervisor Support	1.185*	0.468	0.960*	0.391	1.434***	0.413	4.522***	0.860
Grit × Supervisor Support	−0.346**	0.132	−0.292**	0.110	−0.415***	0.116	−1.325***	0.242
Colleague Support	0.528	0.49	1.301**	0.410	0.239	0.432	−3.681***	0.901
Grit × Colleague Support	−0.123	0.141	−0.330**	0.118	−0.031	0.125	0.913***	0.260
Model Summary	*R*^2^ = .467; *F* (5, 336) = 58.810; *p* < .001		*R*^2^ = .547; *F* (5, 336) = 81.287; *p* < .001		*R*^2^ = .572; *F* (5, 336) = 89.968; *p* < .001		*R*^2^ = .409; *F* (5, 336) = 46.423; *p* < .001	

* *p* < .05.

** *p* < .01.

*** *p* < .001.

**Figure 2:**
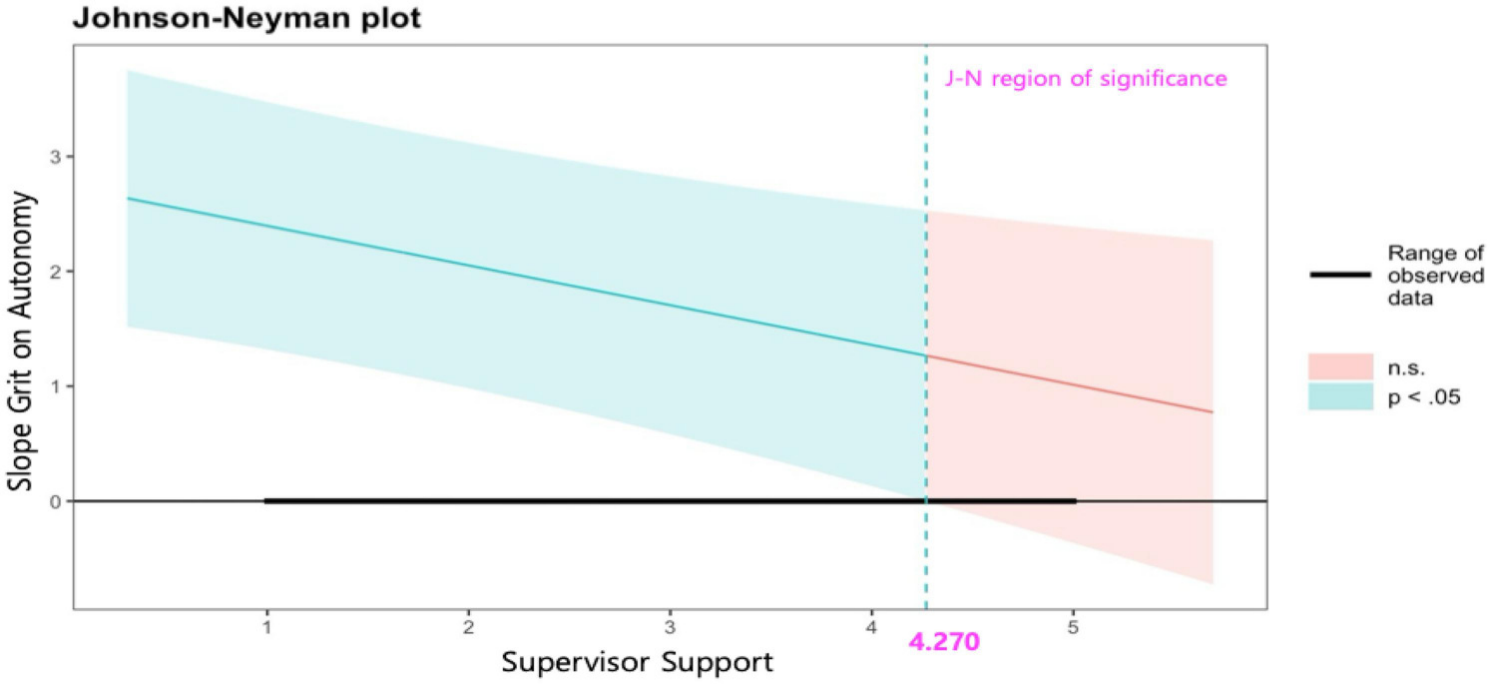
Johnson-Neyman plot on the effects of grit on autonomy by level of supervisor support.

First, grit was found to have a positive effect on autonomy (*b* = 2.742, *p* < .001). The interaction between grit and supervisor support had a significant effect on autonomy (*b* = −0.346, *p* < .01), while the interaction between grit and colleague support was not significant (*b* = −0.123, *p* > .05). Specifically, to clarify the conditional moderating effect of supervisor support, the Johnson-Neyman (J-N) approach was applied. As shown in Figure 2, as supervisor support increased, the effect of grit on autonomy decreased. However, a significant moderating effect was observed from the level of 1 (effect = 2.396, CIs = 1.319–3.473) to the level of 4.270 (effect = 1.265, CIs = 0.000–2.531), but the moderating effect was not significant at levels above 4.400 (effect = 1.220, CIs = −.063–2.504).

**Figure 3:**
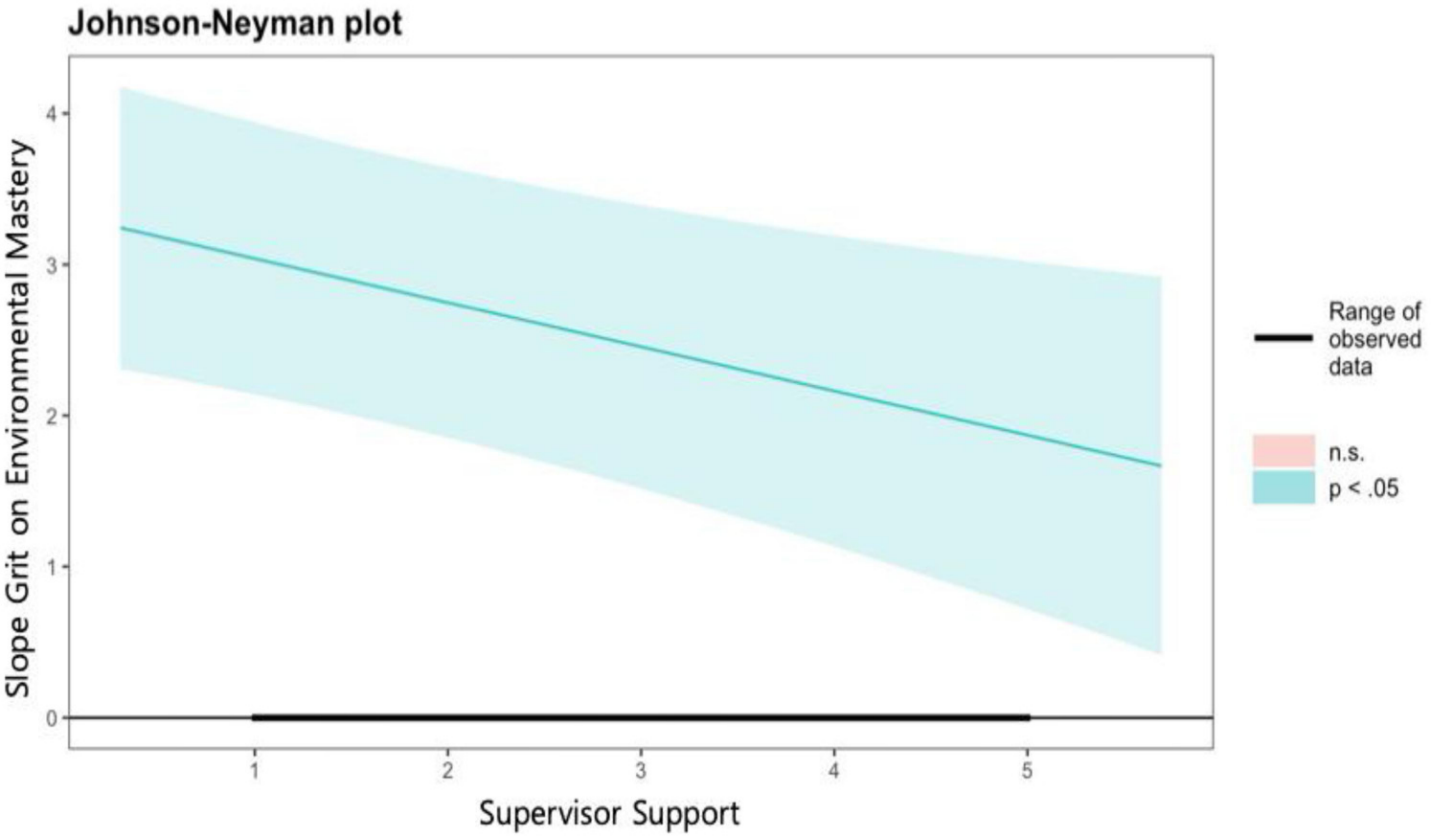
Johnson-Neyman plot on the effects of grit on environmental mastery by level of supervisor support.

**Figure 4:**
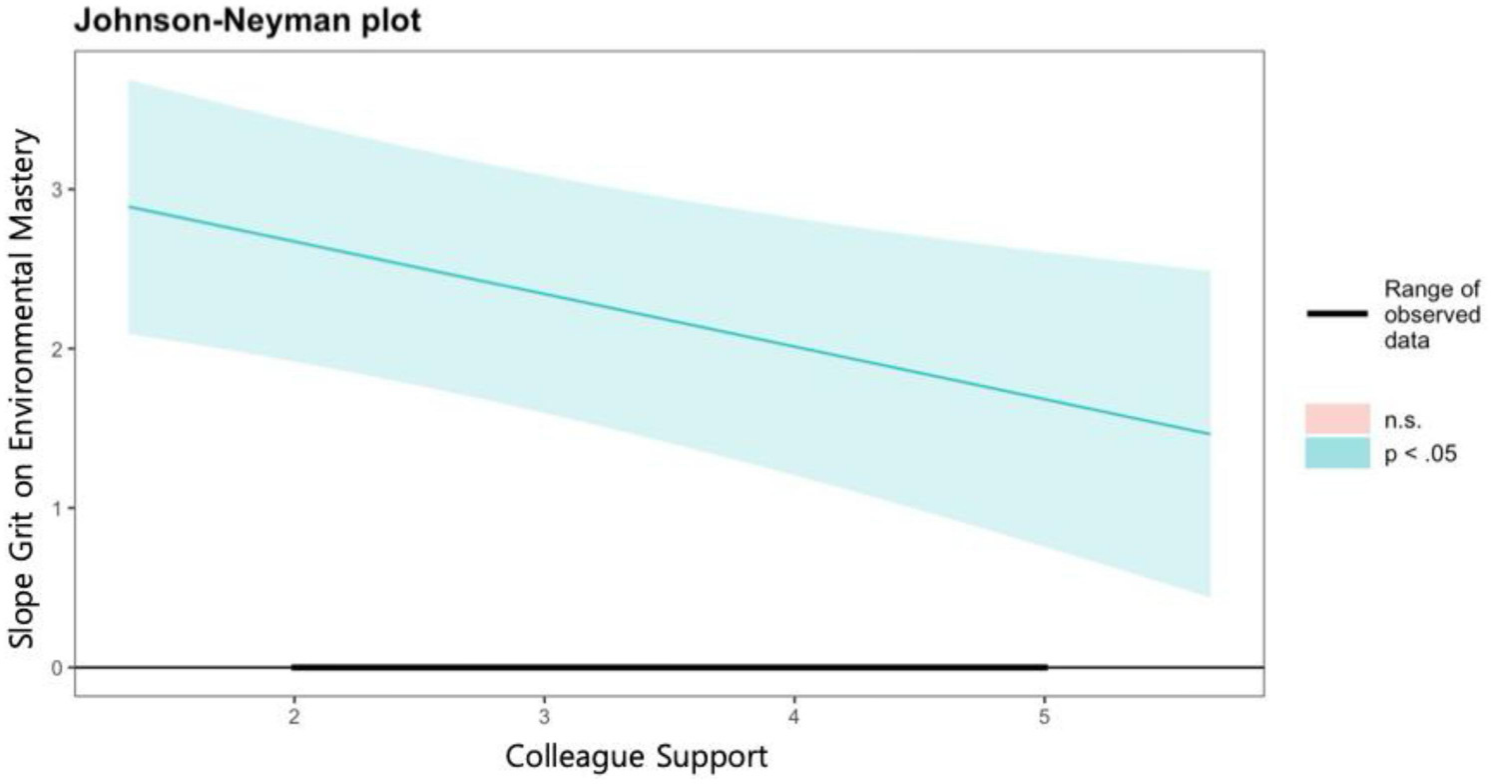
Johnson-Neyman plot on the effects of grit on environmental mastery by level of colleague support.

Second, grit had a significant effect on environmental mastery (*b* = 3.332, *p* < .001), and the two types of social support were shown to moderate the relationship. Specifically, the moderating effects of supervisor support (*b* = −0.292, *p* < .01) and colleague support (*b* = −0.330, *p* < .01) were both negatively significant. As shown in Figures 3 and 4 of the J-N analysis results, the moderating effects of supervisor support and colleague support were significant across all range, and it was observed that the effect of grit on environmental mastery weakened as social support increased.

**Figure 5:**
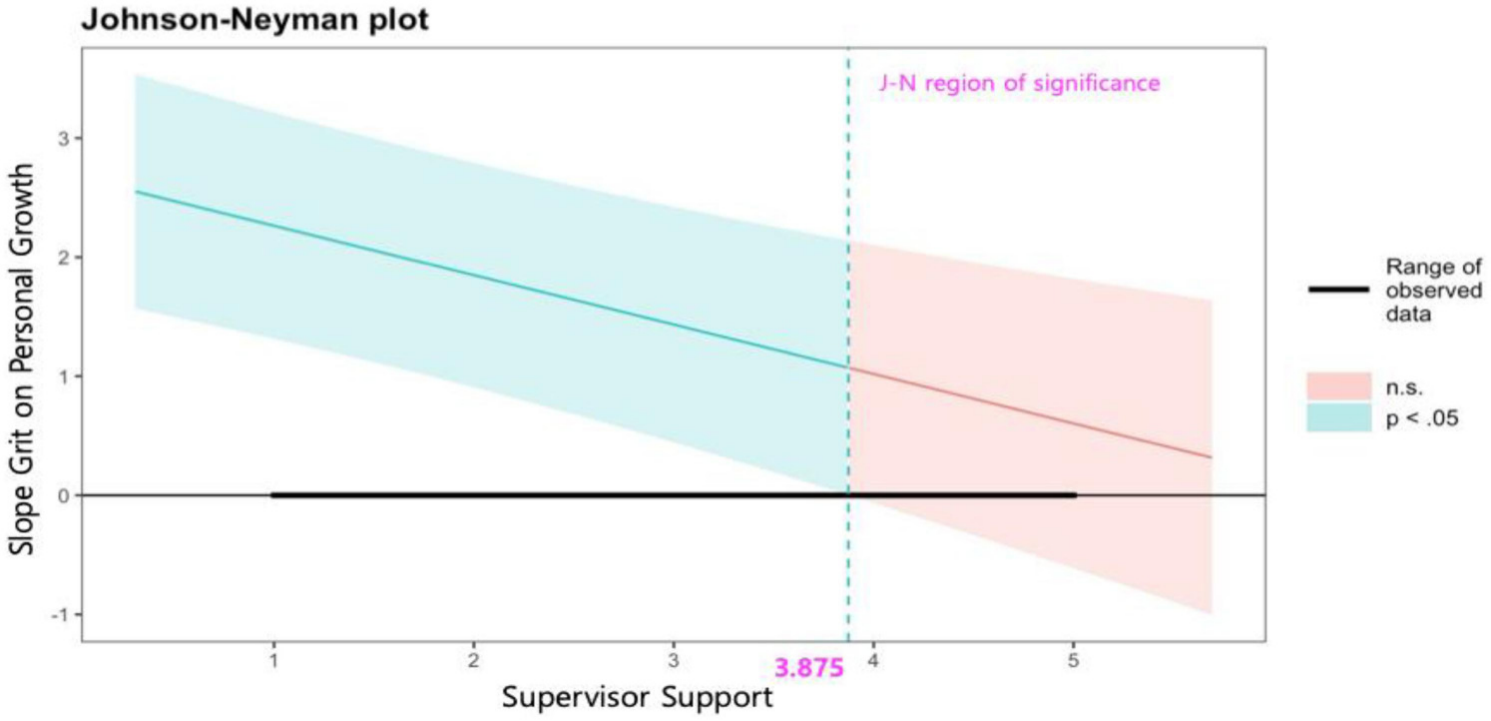
Johnson-Neyman plot on the effects of grit on personal growth by level of supervisor support.

Grit and personal growth were positively associated (*b* = 2.679, *p* < .001), and among the two types of social support, only supervisor support moderated this relationship (*b* = −0.415, *p* < .001). As shown in Figure 5 of the J-N approach results show that as supervisor support increases, the effect of grit on personal growth decreases. However, when supervisor support levels range from 1 to 3.875 (effect = 2.264, CIs = 1.313–3.214; effect = 1.070, CIs = −0.066–2. 102), the effect of grit on personal growth was significant, while it was not significant at levels of 4 or higher (effect = 1.018, CIs = −0.066–2.102).

**Figure 6:**
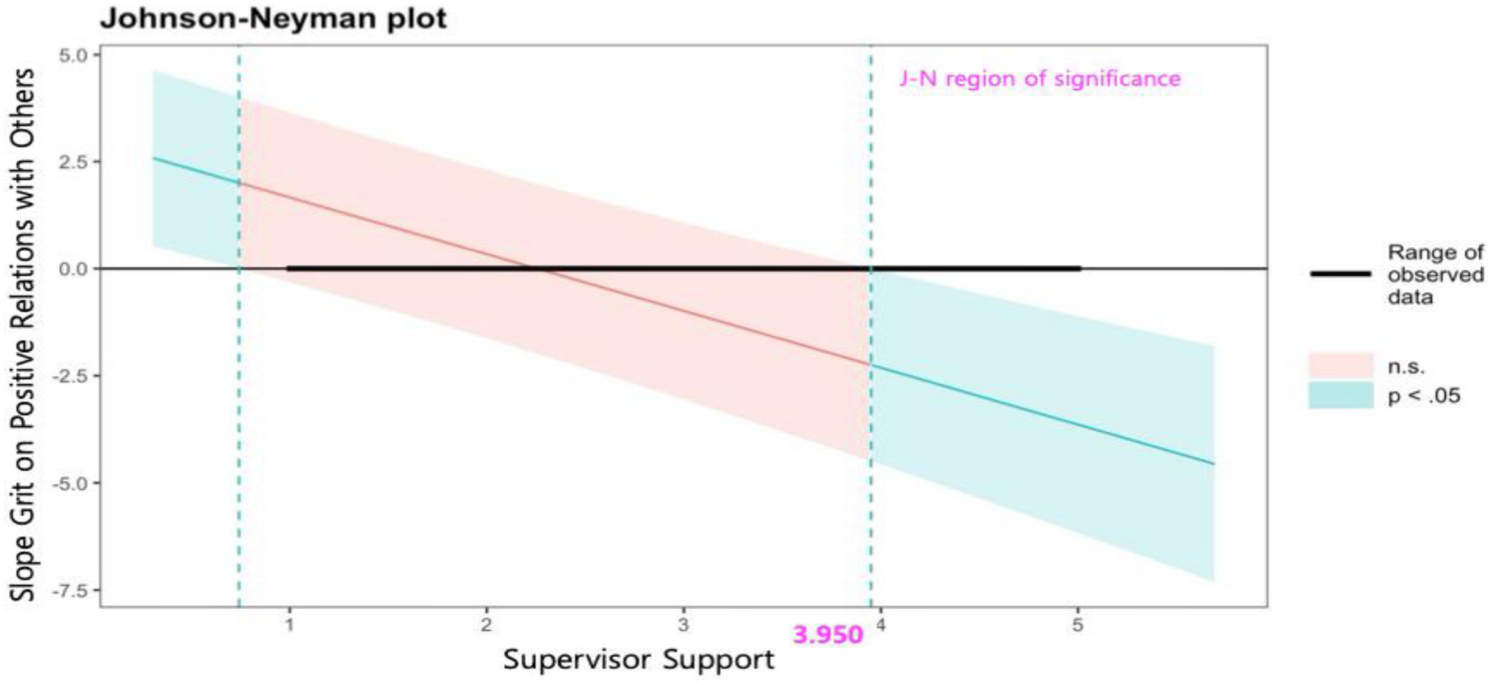
Johnson-Neyman plot on the effects of grit on positive relations with others by level of supervisor support.

**Figure 7:**
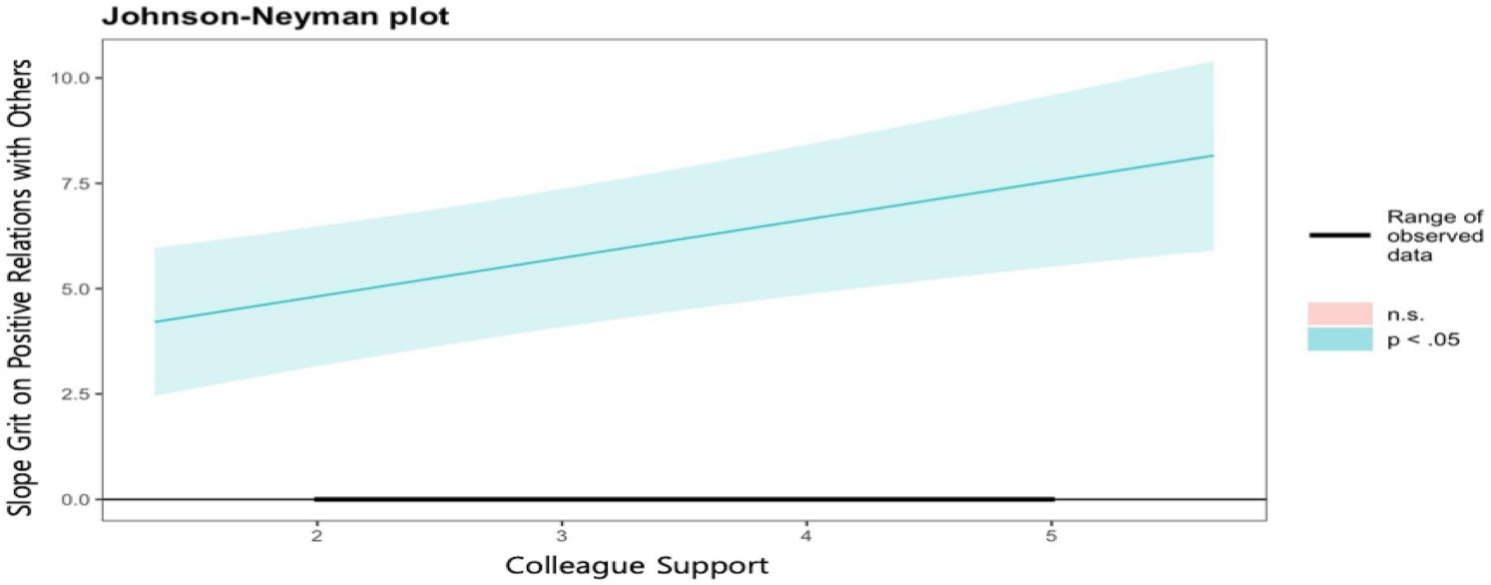
Johnson-Neyman plot on the effects of grit on positive relations with others by level of colleague support.

Finally, grit was positively associated with positive relations with others (*b* = 2.987, *p* < .01). This relationship was moderated by both types of social support. Specifically, the moderating effect of supervisor support was *b* = −1.325 (*p* < .001), and a unique finding from the J-N approach results (see Figure 6) was that this effect was only significant when the level of supervisor support was 4 or higher (effect = −2.579, CIs = −4.573 to −0.054). Furthermore, this effect became stronger in a negative direction as supervisor support increased. Colleague support positively moderated these relationships (*b* = 0.913, *p* < .001). As shown in the results of the J-N approach in Figure 7, the effect of grit on positive relations with others was significant across all range, and this relationship became stronger as colleague support increased. In other words, as colleague support increased, the influence of grit on positive relations with others also increased.

## 4 Discussion

Based on the empirical findings and observed moderation effects, this study offers several actionable recommendations for athletes, coaches, sport psychologists, and institutional stakeholders seeking to enhance psychological well-being in collegiate athletic contexts.

### 4.1 Grit-Focused training for athletes

The present study found that grit was a consistent and significant predictor of four key domains of psychological well-being—autonomy, environmental mastery, personal growth, and positive relations—among collegiate athletes. These results align with recent findings showing that grit contributes to athletes' sustained engagement, adaptive coping, and reduced burnout in competitive contexts (18). From a motivational standpoint, grit reflects a form of internally regulated perseverance that supports the pursuit of self-concordant goals, thereby enhancing satisfaction of the basic psychological needs for autonomy and competence (9). Several studies have highlighted that the benefits of grit are most pronounced when it is expressed within autonomy-supportive environments rather than under controlling or pressuring climates (19, 20). For example, autonomy-supportive feedback has been shown to strengthen athletes' intrinsic motivation and psychological well-being by validating their perspectives and emphasizing self-referenced progress. The current findings extend this evidence by suggesting that grit functions as a stable psychological resource whose adaptive potential depends on the surrounding motivational climate. At the same time, this study adds nuance to previous research by emphasizing that grit should not be treated as a universally positive trait in isolation. When athletes' perseverance is disconnected from social support or self-determined motives, grit may lead to rigid persistence or maladaptive overtraining (21, 38). Thus, rather than promoting grit as a standalone quality, interventions should integrate it within comprehensive training frameworks that foster autonomy, competence, and relatedness. Practically, programs aimed at cultivating grit can include structured strategies such as long-term goal-setting workshops, cognitive-behavioral reflection on setbacks, and narrative sharing that helps athletes reinterpret adversity as opportunities for mastery (22, 23). These approaches align with current psychometric and motivational research showing that grit, when developed in tandem with supportive social contexts, promotes sustainable performance and psychological well-being. In sum, the present findings contribute to a growing body of evidence that situates grit not merely as a personal strength but as a contextually sensitive psychological resource whose effectiveness depends on autonomy-supportive, need-satisfying sport environments.

### 4.2 Supportive coaching behaviors as catalysts

The moderation analysis revealed that perceived coach support significantly influenced the association between grit and psychological well-being, showing an unexpected negative interaction. Specifically, at higher levels of coach support, the positive effects of grit were attenuated across several domains of well-being. This finding contrasts with earlier assumptions that coach support universally enhances athletes' adaptation (24) but aligns with recent evidence suggesting that excessive or controlling support can undermine athletes' autonomy and intrinsic motivation (25, 26). According to Self-Determination Theory, external support promotes well-being only when it satisfies rather than thwarts athletes' basic psychological needs (9). High-grit athletes often exhibit strong self-regulatory skills and may perceive overly directive coaching as controlling, thereby reducing their sense of autonomy and internal motivation. Conversely, low-grit athletes may benefit from greater external structure and feedback to sustain effort. This differential pattern explains the negative moderation observed in the current study. Practically, these results emphasize the need for coaches to adopt flexible, autonomy-supportive behaviors that match athletes' dispositional profiles. Rather than providing uniform encouragement, coaches should offer meaningful choices, acknowledge athletes' perspectives, and encourage self-initiated goal pursuit. Such individualized coaching fosters both persistence and psychological well-being in collegiate sport contexts. Although controlling aspects of coach support were theoretically inferred rather than directly measured in the present study, this interpretation aligns with Self-Determination Theory. Future research should empirically distinguish between autonomy-supportive and controlling coach behaviors to verify these mechanisms.

### 4.3 Teammate support as a synergistic resource

Peer support demonstrated dual moderating effects: it strengthened the positive link between grit and positive relations but attenuated the association between grit and environmental mastery. This nuanced pattern indicates that while supportive teammates amplify the relational benefits of grit, they may reduce the need for self-reliant mastery strategies. These findings are consistent with previous research emphasizing the buffering and facilitative roles of social support in sport (13, 35). Moreover, cross-cultural validation studies (19, 27, 28) have shown that perceived peer support reliably predicts athletes' emotional balance, sense of belonging, and interpersonal growth. The current results extend this literature by demonstrating that peer support interacts with personal traits such as grit to determine specific domains of well-being. From a theoretical perspective, the Stress-Buffering Model (12) and Self-Determination Theory together explain this pattern: peer support not only reduces stress reactivity but also nurtures relatedness, a key psychological need. Thus, the synergy between grit and teammate support reflects a social mechanism that transforms perseverance into meaningful interpersonal engagement. Practically, teams should institutionalize peer-mentorship programs, emphasize cooperative goals, and normalize help-seeking behaviors. Encouraging athletes to both give and receive support fosters a culture where perseverance and connection coexist, reinforcing both performance and well-being.

### 4.4 Institutional infrastructure for embedded support

Beyond interpersonal dynamics, the study underscores the institutional dimension of social support. The moderating influence of coach and peer support indicates that well-being in collegiate sport is shaped not only by individual traits but also by systemic structures. This resonates with recent calls for integrated psychosocial infrastructures in university athletics (29). Institutions that establish cohesive networks among coaches, sport psychologists, trainers, and academic advisors can better address athletes' psychological needs. Evidence from recent validation studies (19, 27) suggests that tools such as the Need Satisfaction and Frustration Scale and the Behavioral Regulation in Sport Questionnaire can serve as diagnostic instruments for monitoring motivational climates. Within Self-Determination Theory, such institutional mechanisms create autonomy-supportive environments that sustain athletes' engagement and well-being. Therefore, social support should be regarded as a fundamental resource, equivalent to physical infrastructure. Program evaluations and coach assessments should include social-support indicators to ensure that athlete development is both performance-driven and psychosocially sustainable.

### 4.5 Personalized athlete development and monitoring

The current findings demonstrate that grit's influence operates within a network of social interactions, implying that athlete development must integrate psychological assessment and contextual awareness. This supports a shift from uniform training models toward personalized monitoring frameworks. Earlier research on coach–athlete dyads (30) and recent cross-national psychometric analyses (26, 28) both highlight the importance of context-sensitive measurement. Consistent with this perspective, the present study indicates that the interplay between grit and social support varies across individuals, suggesting that individualized assessment of both variables is crucial for targeted intervention. In practice, athletic programs can employ validated inventories of grit, resilience, and perceived support to identify athletes' psychological profiles. Low-grit athletes may require structured resilience training and goal-clarification workshops, whereas high-grit but socially isolated athletes may benefit from relational and community-building programs. Personalized monitoring thus translates research insights into actionable sport-psychological strategies that enhance both performance and mental health.

### 4.6 Rethinking “toughness” in sport

The findings challenge the traditional narrative that equates mental toughness with psychological strength. Although grit represents sustained perseverance, the study shows that its benefits are context-dependent and can be diminished in unsupportive or overly controlling environments. Recent conceptual reviews (31, 38) argue that toughness should be reframed as adaptive flexibility—the capacity to persist while also seeking support and regulating effort appropriately. The present results empirically support this shift, indicating that grit combined with social support fosters more sustainable well-being than grit alone. From a theoretical standpoint, integrating Self-Determination Theory with the Stress-Buffering Model (12) suggests that autonomy-supportive relationships transform perseverance into adaptive coping, reducing the psychological costs of intense striving. In applied contexts, coaches and sport institutions should promote a “supported perseverance” culture, where seeking psychological assistance is normalized and valued. Recognizing that optimal performance arises from both persistence and connectedness reframes mental toughness as a collective, not solitary, strength.

## 5 Conclusion

This study provides integrative evidence that grit functions as a central psychological resource that enhances multiple dimensions of well-being—autonomy, environmental mastery, personal growth, and positive relations—among collegiate athletes. However, the positive effects of grit are contingent upon the social environments in which athletes train and compete. Consistent with recent psychometric and motivational research emphasizing autonomy-supportive climates and the accurate assessment of social support (19, 26–28, 32), the findings highlight that interpersonal and institutional contexts significantly modulate the influence of grit. Specifically, while grit enhances psychological well-being, its benefits are amplified through peer support and may be diminished under excessive or controlling coaching behaviors. These results align with the principles of Self-Determination Theory (9, 20), suggesting that perseverance yields optimal outcomes when expressed within autonomy-supportive, need-satisfying environments. The synergy between individual perseverance and socially supportive sport ecosystems thus provides a comprehensive framework for sustainable mental health and adaptive functioning in collegiate athletics. Accordingly, future sport programs and psychological interventions should combine grit-focused personal development with institutional strategies that cultivate supportive, autonomy-enhancing climates—promoting both athletic excellence and long-term psychological resilience.

## 6 Practical implications for coaching practice

The findings highlight that grit should be developed and supported within autonomy-supportive coaching environments. Coaches can apply these insights by encouraging athletes to set self-directed goals, offering feedback that acknowledges their perspectives, and fostering peer collaboration. Such practices not only strengthen athletes' perseverance and motivation but also promote long-term psychological well-being and sustainable performance in collegiate sports.

## 7 Limitations and future directions

This study provides empirical support for the complex interplay between individual traits (such as grit) and environmental factors (social support) in promoting psychological well-being among collegiate athletes. Grit emerged as a robust predictor of four core dimensions of well-being—autonomy, environmental mastery, personal growth, and positive relations. Notably, social support moderated these associations in domain-specific ways: higher levels of coach support attenuated the benefits of grit in certain domains, whereas teammate support amplified grit's positive impact—particularly for interpersonal functioning. These findings underscore the importance of tailoring psychological and coaching strategies to athletes' dispositional profiles rather than relying on one-size-fits-all models. In particular, they call for a shift away from paradigms that glorify mental toughness in isolation toward a more balanced approach that integrates sustained effort with supported perseverance. To translate these insights into practice, sport programs should prioritize the development of comprehensive psychosocial infrastructure. This includes delivering educational and mental-skills programs that account for both individual differences and contextual factors, thereby reinforcing the link between grit and psychological well-being. Such an integrative framework promotes athletic success and fosters long-term mental health and sustainable engagement among collegiate athletes. Despite its valuable contributions, this study is not without limitations.

First, the cross-sectional design precludes causal inferences regarding the relationships among grit, social support, and psychological well-being. Although the results are consistent with theoretical expectations derived from self-determination theory and the stress-buffering model, longitudinal or experimental studies are needed to establish temporal ordering and causal mechanisms. For instance, future research could adopt experimental or intervention-based designs to examine whether enhancing grit or perceived social support directly leads to improvements in well-being outcomes. Such approaches would clarify whether the observed associations represent causal pathways or reflect reciprocal influences among these constructs.

Second, the cultural context of the current study should be considered when interpreting the findings. Because the data were collected exclusively from Korean collegiate athletes, the results may not be fully generalizable to athletes from different cultural backgrounds, particularly those from Western contexts that emphasize individualistic values. Cross-cultural comparisons or studies including control groups from diverse cultures are needed to determine whether the observed relationships between grit, social support, and psychological well-being hold consistently across cultural settings. Future research should therefore examine these constructs within varying cultural frameworks to strengthen the external validity and universality of the present findings.

Third, certain subscales in this study (e.g., grit and autonomy) exhibited relatively low Cronbach's *α* values (*α* = .623 and *α* = .674, respectively). While these values are slightly below the conventional threshold of.70, they remain within an acceptable range for psychological constructs that encompass heterogeneous items or multidimensional attributes (33). Moreover, previous sport psychology studies using similar adapted instruments have reported comparable reliability coefficients among collegiate athlete populations [e.g., (6, 8)]. Given that these subscales demonstrated theoretically consistent correlations and significant predictive relationships in the current analyses, their inclusion was retained to preserve conceptual integrity rather than purely statistical optimization. Nonetheless, future research should consider further refinement or cultural validation of these measures to improve internal consistency and measurement precision.

Fourth, potential effects related to gender and type of sport were not a primary focus of this study. Although the sample included both male and female athletes as well as participants from individual and team sports, these variables were not analyzed as moderators in the present model. *post-hoc* comparisons were conducted, and no significant differences were found in the main study variables across gender or sport type. This suggests that the observed relationships among grit, social support, and psychological well-being were relatively stable across these subgroups.

Nevertheless, recent research indicates that motivational and psychosocial mechanisms can vary depending on gender and sport context (28, 32). These studies emphasize that individual vs. team environments and gender-based socialization processes may shape athletes' perceptions of support and well-being. Future research should therefore examine whether gender and sport type moderate the effects of grit and social support, using larger and more diverse samples to improve the robustness and generalizability of findings.

## Data Availability

The original contributions presented in the study are included in the article/Supplementary Material, further inquiries can be directed to the corresponding author.
